# Complications in neonates of mothers with gestational diabetes mellitus receiving insulin therapy versus dietary regimen

**Published:** 2016-04

**Authors:** Zhaleh Fazel-Sarjoui, Amirali Khodayari Namin, Maryam Kamali, Nazanin Khodayari Namin, Ali Tajik

**Affiliations:** 1 *Department of Gynecology, Jàvaheri Hospital, Islamic Azad University, Tehran Medical Branch, Tehran, Iran.*; 2 *Department of Radiology* *,* * Milad Specialized and Subspecialized Hospital, Tehran, Iran* *.*; 3 *Department of General Surgery, Ayatollah Taleghani Hospital, Shahid Beheshti University of Medical Sciences, Tehran, Iran.*; 4 *Javaheri Hospital, Islamic Azad University, Tehran Medical Branch, Tehran, Iran.*; 5 *Department of Community Medicine, Tehran University of Medical Sciences, Tehran, Iran.*

**Keywords:** *Neonate*, *Gestational diabetes mellitus*, *Treatment*

## Abstract

**Background:**

Gestational diabetes mellitus (GDM) is a common obstetrical complication with both maternal and fetal side effects.

**Objective:**

This study was performed to determine the complications in neonates of mothers with GDM receiving insulin vs. dietary regimen.

**Materials and Methods:**

In this prospective cohort study, 140 neonates of mothers with GDM attending Javaheri Hospital of Azad University in Tehran in 2013 and 2014 were enrolled and the complications in those receiving insulin versus. dietary regimen were compared.

**Results:**

The results demonstrated that 95.7% of those who received a dietary regimen and 85.7% among those who received insulin had a good outcome showing statistically significant differences (p=0.042). The mortality rate was not differed among the patients in two groups (p>0.05).

**Conclusion:**

According to the results of this study, it may be concluded that the frequency of complications in neonates of cases with GDM is getting less by receiving dietary regimen.

## Introduction

Gestational Diabetes Mellitus (GDM) is a common complication of pregnancy resulting from environmental and genetic factors (1). Beside numerous fetal-maternal complications, it may result in increased probability of type 2 diabetes and other metabolic diseases in mother after pregnancy (2, 3). It necessitates the diagnosis and treatment of disease and contributing factors (3). Additionally, infants of diabetic mothers would develop further problems such as large for gestational age and metabolic disorders resulting in need to further care leading to higher health care costs (4).

Since the main pathophysiological mechanism of GDM is insulin resistance, GDM treatment is mainly with insulin (5). But in thin cases with mild hyperglycemia non- insulin methods, including dietary regimens may be used (5, 6). Also, oral anti-diabetic agents may be used which generally have the same or occasionally better results compared with insulin (7, 8). However, regarding the critical situation in pregnancy and for reduction of maternal and fetal side effects, less use of drugs and more use of dietary regimens is rational. But at first, the efficacy of dietary regimen should be certified. Hence, this study was performed to determine the complications in neonates of mothers with GDM receiving insulin versus dietary regimen.

## Materials and methods

In this prospective study, 140 consecutive neonates of mothers with GDM attending Javaheri Hospital of Azad University in Tehran in 2013 and 2014 were enrolled. The inclusion criteria were aging from 20-35 years and lack of congenital anomaly. The current study was approved by the Ethical Committee of Islamic Azad University, Tehran Medical Branch and Javaheri Hospital, and written informed consents were obtained before the study performance.

The exclusion criteria were aging less than 20 and more than 35 years and presence of congenital anomaly. The gravid (previous pregnancy times with more than 20 weeks passed from gestational age) was assessed without range limitation. The complications in those women who were receiving 20 units of single-dose short-acting insulin were compared with those who were using dietary regimen including six meals (three main and three brief meals) with daily calorie intake between 1200 and 1800 kilocalories. The complications included intrauterine growth retardation, preterm labour, low birth weight, and macrosomia.

**Statistical analysis**

Data analysis was performed among 140 subjects, including 70 neonates and their mothers in insulin group and 70 subjects in the dietary regimen group, which was done by SPSS (version 13.0) software [Statistical Procedures for Social Sciences, Chicago, Illinois, USA]. Chi-Square, Fisher, and independent sample T-test were used and were considered statistically significant at p<0.05.

## Results

As shown in table I, the mean maternal age in insulin and regimen groups were 30.1±5.1 and 29.1±4.6 years, respectively (p>0.05). Mean neonatal weight in insulin and regimen groups were 3147.0±297.5 and 3247.6±660.8 gr, respectively (p>0.05). Gravid more than one was seen in 68.6% and 44.3% in insulin and regimen groups (p>0.05). Male sex was seen in 68.6% and 70.0% in insulin and regimen groups (p>0.05). Term neonates were more present in regimen group vs. insulin group (100% vs. 64.3%). However, the mortality rate was similar between groups (p>0.05); good outcomes (without complications including intrauterine growth retardation, preterm labour, low birth weight, and macrosomia) were more seen in dietary regimen group (Table II).

**Table I:** Demographic characteristics of patients in two groups

**Variable**	**Group**s		**p-value**
	**Insulin**	**Dietary Regimen**	
Age (year)	30.1±5.1	29.1±4.6	> 0.05
Weight (gram)	3147.0±297.5	3247.6±660.8	> 0.05
FBS (mg/dl)	114.5±34.3	118.1±42.6	> 0.05
2-Hour BS (mg/dl)	175.3±40.2	177.9±38.2	> 0.05
4-Hour BS (mg/dl)	144.7±23.8	147.2±19.5	> 0.05
Pregestational Weight (kg)	62.2±8.4	61.9±8.2	> 0.05
Weight increase during pregnancy (kg)	9.9±2.5	10.4±2.9	> 0.05

**Table II:** Outcomes and mortality rate in two groups

**Index**	**Groups**	
	**Insulin**	**Dietary Regimen**
Complications*	10 (14.3%)	3 (4.3%)
Mortality*	1 (1.4%)	-----

* p< 0.05

## Discussion

This study was performed to determine the frequency of complications in neonates of mothers with GDM receiving insulin therapy versus dietary regimen. Our results demonstrated that 95.7% of those who received a dietary regimen and 85.7% among those who received insulin therapy had a better outcome (without complications) showing significant differences (p=0. 042). But mortality rate was not differed between the groups (p>0.05). Control of maternal blood sugar with dietary recommendations, and close monitoring of blood glucose levels and the treatment with insulin, if required, has been shown to decrease fetal and maternal morbidities, but has not been established for dietary regimens (9). Diet and exercise interventions are useful for prevention of gestational diabetes mellitus (10). Surprisingly, even frequent use of fried food in pregestational phase is significantly associated with a greater risk of gestational diabetes mellitus (11).

The study by Hernandez *et al*, revealed that a higher- complex carbohydrate diet in women with gestational diabetes mellitus would result in better glucose control (12). Philipson *et al*, reported that large for gestational age (LGA) neonates were born more frequently from mothers who were received insulin therapy versus dietary regimen, but the mortality rate was not differed like our study (13). Giuffrida and colleagues conversely reported outcomes that there are no statistically significant differences between those who were received insulin therapy and subjects on a dietary regimen (14). 

In study by Mello et al, the poor post labor outcomes, including large for gestational age (LGA) neonates were reported with lower rate in subjects receiving dietary regimen alone versus those who received dietary regimen plus insulin therapy, like our findings (15). Langer and colleagues reported no difference effects between oral anti-diabetic medications versus insulin therapy (16). Garner *et al* reported that non-drug subjects vs. drug treatments patients have not been differed for fetal outcome, which leads to recommend using non- drug care (17). 

According to our novel findings, less neonatal complications are seen in cases with GDM receiving dietary regimen. Therefore, the best supplementary treatment, beside conventional medications such as insulin in GDM cases could be dietary regimen and if it is not effective the other supplementary treatments should be used. However, further studies with larger sample size and comparing of other optional treatments should be performed. 
